# Deep Multimodal Fusion Autoencoder for Saliency Prediction of RGB-D Images

**DOI:** 10.1155/2021/6610997

**Published:** 2021-05-05

**Authors:** Kengda Huang, Wujie Zhou, Meixin Fang

**Affiliations:** ^1^School of Information and Electronic Engineering, Zhejiang University of Science & Technology, Hangzhou 310023, China; ^2^Institute of Information and Communication Engineering, Zhejiang University, Hangzhou 310027, China

## Abstract

In recent years, the prediction of salient regions in RGB-D images has become a focus of research. Compared to its RGB counterpart, the saliency prediction of RGB-D images is more challenging. In this study, we propose a novel deep multimodal fusion autoencoder for the saliency prediction of RGB-D images. The core trainable autoencoder of the RGB-D saliency prediction model employs two raw modalities (RGB and depth/disparity information) as inputs and their corresponding eye-fixation attributes as labels. The autoencoder comprises four main networks: color channel network, disparity channel network, feature concatenated network, and feature learning network. The autoencoder can mine the complex relationship and make the utmost of the complementary characteristics between both color and disparity cues. Finally, the saliency map is predicted via a feature combination subnetwork, which combines the deep features extracted from a prior learning and convolutional feature learning subnetworks. We compare the proposed autoencoder with other saliency prediction models on two publicly available benchmark datasets. The results demonstrate that the proposed autoencoder outperforms these models by a significant margin.

## 1. Introduction

With the rapid development in the consumer electronic industry, various RGB-D applications and services have become increasingly popular for enhanced user experience [1–6]. The RGB-D image processing technologies for RGB-D applications and services can be further improved by developing better models of RGB-D perception [7–10]. However, predicting the saliency in RGB-D images is a particularly intractable problem [11–16]. Nevertheless, it is promising, as it can certainly help in visually improving approaches such as video coding [17], image quality measurement [18–21], visual comfort prediction [22–24], and image retargeting [25, 26].

In the last two decades, many saliency prediction methods for RGB images have been significantly improved, and numerous models have been proposed [27–37]. For example, Itti et al. presented a saliency prediction metric for RGB image by using a biologically plausible neural architecture, whereby hand-designed low-level visual features could be extracted from intensity, orientation, and color [27]. Later, Hou and Zhang presented a saliency prediction model based on transform domain [28]. Harel et al. proposed a graph-based visual saliency (GBVS) prediction metric [29]. Fang et al. introduced a saliency prediction model based on the biological visual system (BVS) and the amplitude spectrum [30]. Zhang et al. presented a simple saliency prediction approach, namely, SDSP, by integrating three prior maps [31]. Other relevant works can be found elsewhere [32–37].

Most previous studies employed human-designed mechanisms to compute hand-designed low-level visual features, which do not sufficiently obtain the high-level semantic structural information that can help in saliency prediction. Moreover, it would be insufficient to handle large-scale data with complex distributions. As deep architectures were primarily inspired by biologically simulated neural networks, it would be appropriate to establish a computational framework of saliency prediction using deep architecture. Currently, with the recent advancements in deep convolutional neural networks (CNNs), RGB image saliency prediction has improved considerably in comparison to using conventional nondeep learning techniques. Vig et al. proposed the ensemble of deep networks (eDNs), which is an early deep architecture that automatically learns the bio-inspired hierarchical features to predict RGB image saliency [38]. Kümmerer et al. proposed DeepGaze I [39] and DeepGaze II [40] using feature representations from the existing pretrained AlexNet [41] and VGGNet [42], respectively. Li and Yu utilized nested windows as inputs to extracted multiscale CNNs features and later integrated them to generate a saliency map [43]. Liu et al. proposed a deep architecture for RGB image saliency prediction using multiresolution CNNs that learn both low-level saliency cues and high-level factors [44]. Huang et al. proposed an architecture including a deep CNN applied to two scales [45]. They compared CNN architectures of different standards, such as AlexNet [41], VGGNet [42], and GoogLeNet [46], and demonstrated the effectiveness of their architecture, particularly the one based on the VGGNet. Thereafter, several VGGNet based saliency prediction models have been proposed [47–57]. The aforementioned deep-learning-based saliency prediction models have achieved promising results. However, these models are probably not very effective in predicting the saliency maps of RGB-D images because the feature representations in the models cannot adequately simulate the binocular visual mechanism.

Owing to the fact that the saliency prediction methods for RGB-D images are relatively less developed, little progress has been made. For instance, Wang et al. proposed a depth saliency-based RGB-D saliency prediction model that combines the resulting depth saliency map with an existing RGB saliency prediction model using two methods [58]. Fang et al. introduced an RGB-D saliency prediction model, where all the feature maps were extracted from discrete cosine transformation (DCT) coefficients, which were combined for the final saliency map [59]. Jiang et al. proposed a visual comfort-guided 3D saliency prediction model that not only considers the factors from depth perception but also investigates visual discomfort in the prediction model [60]. Moreover, Qi et al. presented an RGB-D saliency prediction model by combining a texture saliency map, a depth saliency map, and an RGB saliency map using a linear pooling strategy. [61]. In these saliency prediction models, they mainly calculate the saliency map of RGB-D images by simply combining the depth feature map, RGB saliency map, and other factors. Therefore, the performances are limited. Several data-driven approaches have been proposed, wherein machine learning techniques have been used for saliency prediction. Ma and Huang presented a learning-based RGB-D saliency prediction model that includes the depth map and its derived features [62]. Fang et al. proposed an RGB-D saliency prediction model that collects various low-level visual features and combines them using the support vector regression (SVR) [63]. As deep learning-based saliency prediction methods have achieved significant results for RGB images, researchers have been trying to apply these techniques to RGB-D images. Zhang et al. introduced an RGB-D image saliency prediction model based on deep learning techniques. They used AlexNet to extract the color and depth (high-level) features and then combined these to obtain the RGB-D saliency information [64]. However, this model is not learned in an end-to-end deep supervision mean and only uses pretrained AlexNet in extracting the color and depth features from the images. Therefore, the performance is limited.

Deep architecture, the design of which was originally inspired by the functioning of cells in the visual neurons, can be used to obtain various rich features in a hierarchical pattern. In this work, we propose novel CNNs for RGB-D image saliency prediction in a deep supervision manner. The proposed autoencoder comprises four main networks: a color channel network, a disparity channel network, a feature concatenated network, and a feature learning network. The autoencoder ensures that the networks are trainable in an end-to-end deep design and can automatically learn their own priors from training data. The results indicate that the proposed deep autoencoder, by incorporating a disparity channel network and a prior learning subnetwork, helps significantly improve the prediction performance.

  In summary, the following are the three main contributions of this work:

1. The core trainable network of the proposed RGB-D saliency prediction model employs raw RGB-D images as inputs and their corresponding eye-fixation attributes as labels. The model comprises four main networks: a color channel network, a disparity channel network, a feature concatenated network, and a feature learning network. These networks can mine the complex relationship and make the utmost of the complementary characteristics between both color and disparity cues.
2. We introduce a novel deep multimodal fusion autoencoder that sequentially enhances the predicted saliency maps. To the best of the authors' knowledge, our proposed deep autoencoder is a novel end-to-end deep multimodal fusion autoencoder trained and tested for the saliency prediction of RGB-D images on two publicly available datasets.
3. The results indicate that the proposed deep autoencoder, by incorporating a disparity channel network and learned priors, helps significantly improve the prediction performance and achieve outstanding results with competitive generalization properties.

## 2. Proposed Autoencoder

**Figure 1:**
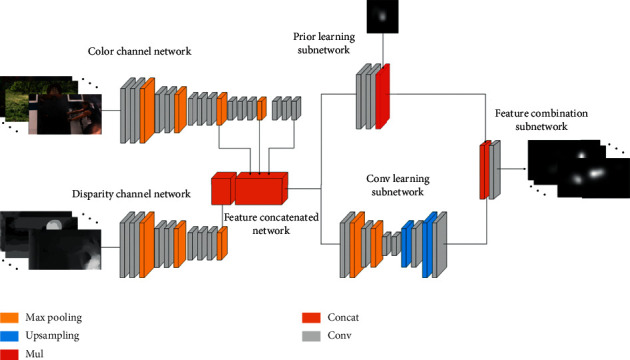
The architecture of the proposed autoencoder.

Figure 1 shows a detailed description of the proposed RGB-D saliency prediction model. The model comprises four main networks: a color channel network, a disparity channel network, a feature concatenated network, and a feature learning network. We first briefly review the four networks and show their mechanisms in predicting the saliency maps of RGB-D images.

### 2.1. Color Channel Network

The color channel network of the proposed deep autoencoder is a CNN with five convolution blocks. This network takes an input color RGB image and outputs the resultant feature maps for the feature concatenated network.

We establish the color channel network based on the standardized 16-layer network from VGGNet [42]. In this study, we consider only convolution blocks and remove fully connected layers. To be more specific, the first two blocks include two convolutional layers each, whereas the subsequent three blocks include three convolutional layers each. If we denote the input feature map as *X*, whose convolution filters are decided by the trainable kernel weight matrix *W*_*s*_ and the trainable bias term vector *b*_*s*_, then the resultant feature map *f*_*s*_ can be obtained as follows:

(1) $$\begin{array}{c} f_{s}\left(X;W_{s},b\right)=W_{s}{*}_{s}X+b_{s}, \end{array}$$

where *∗*_*s*_ denotes the convolution computation with stride *s*. Each convolution layer in the five convolution blocks is restricted to a 3 × 3 convolutional kernel and operates with a downsampling stride of 1. The small convolutional kernels allow the convolution filter to have a highly deep architecture with a lower storage requirement while making the model more discriminative. All the convolutional layers in the autoencoder are followed by point-wise nonlinearity (*e.g*., rectified linear unit (ReLU)) owing to its superior effectiveness and efficiency:

(2) $$\begin{array}{c} \text{ReLU}\left(f_{s}\right)=\left\{\begin{array}{cc} 0, & if\;f_{s}<0,\\ f_{s}, & if\;f_{s}\geq 0. \end{array}\right. \end{array}$$

Furthermore, to improve the translation invariance and representation capability, all the convolution blocks in the VGGNet are often followed by downsampling (*e.g.*, max-pooling) with a kernel pooling size of 2 × 2 and a downsampling stride of 2. For an input RGB image with a spatial resolution of *W* × *H*, the spatial resolution of the resultant feature map will be [*W*/8] × [*W*/8]. Thus, a CNN based on the VGGNet would output a resultant feature map downsampled by a factor of 8. To maintain the spatial information, we omit the last max-pooling layer, while keeping its stride unchanged. Thus, the resultant feature maps of the color channel network are downsampled by a factor of 8 compared to the input. Starting at the first convolution block, the channel dimension in the outputs of each convolution blocks is slowly increased as 64 ⟶ 128 ⟶ 256 ⟶ 512 ⟶ 512. This renders the color channel network to obtain rich structural information of the inputs.

### 2.2. Disparity Channel Network

The disparity/depth information in actual RGB-D environments is crucial to BVS but has been usually underutilized in conventional RGB-D saliency prediction models. Therefore, it is necessary to establish effective and efficient RGB-D saliency prediction models by leveraging the disparity/depth information. In this work, the disparity channel network of the proposed deep autoencoder, which is identical in architecture to VGGNet, is a network with only three convolution blocks. This network takes the input disparity/depth map and outputs feature maps for the feature concatenated network.

Similar to the color channel network, we build the disparity channel network on the standardized 16-layer network from VGGNet [42]. We consider only the first three convolution blocks and remove the rest. The first two convolution blocks contain two convolutional layers each, whereas the subsequent block has three convolutional layers. The convolution blocks end with a pooling layer, and each convolutional layer in the network is followed by an ReLU activity function. In the disparity channel network, there are three pooling layers with a kernel pooling size of 2 × 2 and a stride of 2. For an input disparity map with a spatial resolution of *W* × *H*, the spatial resolution of the resultant feature map will be [*W*/8] × [*W*/8]. Thus, the resultant feature maps of the disparity channel network are downsampled by a factor of 8 compared to the input. Starting at the first convolution block, the channel dimension in the outputs of each convolution blocks is slowly increased as 64 ⟶ 128 ⟶ 256.

### 2.3. Feature Concatenated Network

We first take the resultant feature maps from three different positions of the color channel network: the third max-pooling layer (256 resultant maps), the last convolution block (512 resultant maps), and the last max-pooling layer (512 resultant maps). We then take another set of resultant maps from the last max-pooling layer (256 resultant maps) of the disparity channel network. The various resultant maps can be concatenated to obtain a tensor with 1536 resultant maps. The resulting tensor is then fed through a feature learning network to acquire the RGB-D predicted saliency map.

### 2.4. Feature Learning Network

The feature learning network comprises three subnetworks: a prior learning subnetwork, a convolutional feature learning subnetwork, and a feature combination subnetwork.

(1) Prior learning subnetwork: First, we obtain high-level feature maps by convolving (two convolutional layers with a kernel size of 3 × 3 and a downsampling stride of 1) the output maps of the feature concatenated network. The channel dimension in the output map of the convolution filters is gradually reduced as 320 ⟶ 1. The ReLU activity function is used in all the convolutional layers. Subsequently, we construct a prior learning layer that can learn its own center prior without using the hand-designed prior maps. Toward this end, we learn a rough mask of size *w*_0_ × *h*_0_, initialize it to one, bilinearly upsample it, and apply it to the high-level feature maps with multiplication. Given the entire prior map *O* with a spatial resolution of *w*_0_ × *h*_0_, the pixel values of *O* are interpolated to obtain a learned prior map *P* of size *w* × *h*. We calculate a sampling grid *U* of size *w*_0_ × *h*_0_, associating *O* with real coordinates into *P.* If *U*_*i,j*_ = (*x*_*i,j*_, *y*_*i,j*_), then *O*_*i,j*_ is equivalent to *P* at (*x*_*i,j*_, *y*_*i,j*_); however, as (*x*_*i,j*_, *y*_*i,j*_) are coordinates, we can convolve these and set the following:
  (3) $$\begin{array}{c} V_{x,y}=\sum_{i=1}^{w_{0}}\sum_{j=1}^{h_{0}}Y_{i,j}k_{x}\left(x-x_{i,j}\right)k_{y}\left(y-y_{i,j}\right), \end{array}$$
where *k*_*x*_(*∗*) and *k*_*y*_(*∗*) denote bilinear kernels, *k*_*y*_(*b*)=max(0, (*h*/*h*_0_) − |*b*|), and *k*_*x*_(*b*)=max(0, (*w*/*w*_0_) − |*b*|). *h*_0_ and *w*_0_ are set to [*h*/10] and [*w*/10], respectively, in the experiments.
(2) Convolutional feature learning subnetwork: The convolutional feature learning subnetwork works in a convolutional encoder-decoder model. The encoder part obtains feature maps by convolving (three convolutional layers with a convolutional kernel size of 3 × 3 and a downsampling stride of 1) and downsampling (two pooling layers with a pooling size of 2 × 2 and a downsampling stride of 2) the output maps of the feature concatenated network. Thus, the resultant maps are downsampled by a factor of 4 compared to the input. The decoder part obtains the feature maps by convolving (three convolutional layers with a convolutional kernel size of 3 × 3 and a downsampling stride of 1) and upsampling (two deconvolution layers with kernel size of 3 × 3 and an upsampling stride of 2) the output maps of the encoder part and then outputs with a resolution same as that of the input. Again, the ReLU activity function is employed in all the convolutional layers. The channel dimension in all the convolutional feature learning subnetworks is set as 64.
(3) Feature combination subnetwork: We take the resultant maps from two subnetworks: the output of the prior learning subnetwork and the output of the convolutional feature learning subnetwork. The feature maps have equal dimension and can be concatenated to obtain a tensor. Finally, the output from the feature combination subnetwork is fed to a convolutional layer with one filter and ReLU activity function, the output of which is considered the final saliency map with a spatial dimension of [*W*/8] × [*W*/8] because the downsampling strides in the pooling layers of the first three convolution blocks are greater than unity. We upsample this map to obtain the predicted saliency map with the original size using bicubic interpolation.

To generalize the model and to avoid overfitting, the dropout (a dropout rate of 0.5) is introduced in the output of the feature combination subnetwork.

### 2.5. Training and Testing

The proposed deep autoencoder is executed using the *Keras* deep learning framework. During training, the parameters (e.g., weights and bias) of the color and disparity channel networks are initialized from the pretrained VGGNet [42], whereas the other parameters can be initialized from a standard deviation (SD) of 0.01 and zero mean Gaussian distribution. The autoencoder is encouraged to minimize the values of loss function in the training procedure through Stochastic Gradient Descent (SGD) using back-propagation. The loss function is inspired by one objective: the predicted saliency map should be similar to the corresponding ground-truth saliency density map. Therefore, mean squared error (MSE) or Euclidean distance is a reasonable choice for the evaluation. The overall loss function can be expressed as follows:

(4) $$\begin{array}{c} L_{\text{MSE}}=\frac{1}{M}\sum_{j=1}^{M}{\left(S_{j}-G_{j}\right)}^{\;2}, \end{array}$$

where *S*_*j*_ denotes the *j*^th^ predicted saliency map and *G*_*j*_ denotes the *j*^th^ saliency density map. A mini batch of 8 color and disparity pairs is applied in each iteration. The SGD is applied with a Nesterov momentum of 9 × 10^−1^, a weight decay of 5 × 10^−4^, and a polynomial learning policy with a learning rate of 10^−3^.

During testing, the RGB-D saliency map can be obtained from the feature combination subnetwork. The processing speed of the model is as fast as 0.1 s per RGB-D image, which is conducted on a PC with an 1080Ti GPU and 8 GB of RAM.

## 3. Experimental Results

### 3.1. Datasets

To evaluate the superior performance of our deep autoencoder, two publicly available benchmark datasets were utilized: the NUS-3D Saliency dataset (denoted as NUS) [65] and the NCTU-3D Fixation dataset (denoted as NCTU) [62]. Detailed information of the benchmark datasets is summed up as follows.

The NUS includes 600 RGB-D images including indoor and outdoor scenes. The color stimuli provide a diverse and comprehensive understanding of RGB-D visual scenes for eye tracking analyses. The ground-truth saliency density map was constructed from the human fixations of 80 participants. The age of the participants ranged from 20 to 33 years. Among them, 54 were males and 26 were females.

The NCTU is a collection of 475 RGB-D images along with their raw depth maps and human eye-fixation data. RGB-D content mainly comes from existing RGB-D movies or videos. The depth maps in the dataset were obtained from a Kinect depth sensor. The ground-truth saliency density maps were obtained from 16 subjects using a Tobii TX300 eye tracker, and each RGB-D image stimulus was presented for 4 s.

Following the existing common processing methods [1, 2, 8], the proposed autoencoder requires a train–test procedure. Therefore, in each train–test procedure, 80% was for training, and the remaining was for testing. To ensure robustness of the proposed model, multiple iterations were executed by applying the randomly divided training and testing samples; the median predictions of the indicators from 100 training and testing operations were chosen as the experimental results.

### 3.2. Evaluation Criteria

There are several methods of evaluating the agreement between the fixation density map and the predicted saliency map. Previous works on criteria [66] indicate that it is difficult to obtain an equity comparison for assessing saliency prediction models using one criterion. Here, four widely accepted and known standard evaluation criteria were used to quantitatively compare the fixation density map and the predicted saliency map, namely, Pearson's correlation coefficient (CC), area under the receiver operating characteristic (ROC) curve (AUC), Kullback–Leibler divergence (KLDiv), and normalized scanpath saliency (NSS). For simplicity, we denote the saliency density map as *G*, the binary fixation map as *Q*, and the predicted saliency map as *S*. Then, we illustrate the evaluation criteria in detail.

(1) CC: The CC is a statistical criterion used to determine the level of linear correlation or dependency between two distributions (*S* and *G*).
  (5) $$\begin{array}{c} \text{CC}=\frac{\sigma \left(S,G\right)}{\sigma \left(S\right)\times \sigma \left(G\right)}, \end{array}$$
where *σ*(*S*, *G*) denotes the covariance of *G* and *S*, ranging between −1 and +1, and *σ*(*G*) and *σ*(*S*) denote the SDs of *S* and *G*, respectively. A value closer to −1 or +1 indicates a good agreement between the two saliency maps.
(2) AUC: The AUC criteria are extensively utilized to assess the predicted maps obtained using saliency prediction models. Given an image and its corresponding ground-truth binary fixation map *Q*, the nonfixation and fixation regions can be viewed as negative and positive parts, respectively. The predicted saliency map is then binarily categorized into nonfixation points and fixation points at various thresholds. Through altering the threshold between 0 and 1, the ROC curve is acquired by plotting the false positive rate against the true positive rate, with the area below the curve computed as the AUC value.
(3) KLDiv: The KLDiv assesses the information loss when the distribution *S* is utilized to approximate the distribution *G*, thus making a probabilistic interpretation of *S* and *G*. The KLDiv for *S* and *G* can be expressed as follows:
  (6) $$\begin{array}{c} \text{KLDiv}\left(S\;\left\|\;G\right.\right)=\sum_{i}G_{i}\log\left(\frac{S_{i}}{G_{i}+\varepsilon }+\varepsilon \right), \end{array}$$
where *i* represents the *i*^th^ pixel and *ɛ* denotes a regularization term. The KLDiv is a dissimilarity criterion, and a lower score shows a better approximation of *G* with *S*.
(4) NSS: The NSS is a criterion specifically defined for the evaluation of saliency prediction models. For *S* and *Q*, we have the following relationship:
  (7) $$\begin{array}{c} \text{NSS}=\frac{1}{\beta }\sum_{i=1}^{N}\bar{S}\left(i\right)\times Q\left(i\right),\\ \text{where }\beta =\sum_{i}Q\left(i\right)\text{ and }\bar{S}=\frac{S-\mu \left(S\right)}{\sigma \left(S\right)}, \end{array}$$
where *β* denotes the total number of fixated pixels and *μ* (*S*) represents the mean of *S*.

### 3.3. Comparison of State of the Art

To evaluate the efficiency and effectiveness of our deep autoencoder, we performed a quantitative and qualitative evaluation by comparing it to eight models on the NUS and NCTU datasets, namely, Itti et al. [27], GBVS [29], QFT [30], Wang et al. [58], Fang et al. [59], DeepFix [47], ML-net [51], and DVA [57]. These saliency prediction models have been introduced and have been extensively utilized for comparison. We use the recommended parameter settings provided by the authors.  Table 1 lists the quantitative comparison results on the NUS and NCTU datasets in terms of the CC, KLDiv, NSS, and AUC. From the table, the proposed autoencoder outperforms the rest by a significant margin, thus verifying its robustness and generality.

**Figure 2:**
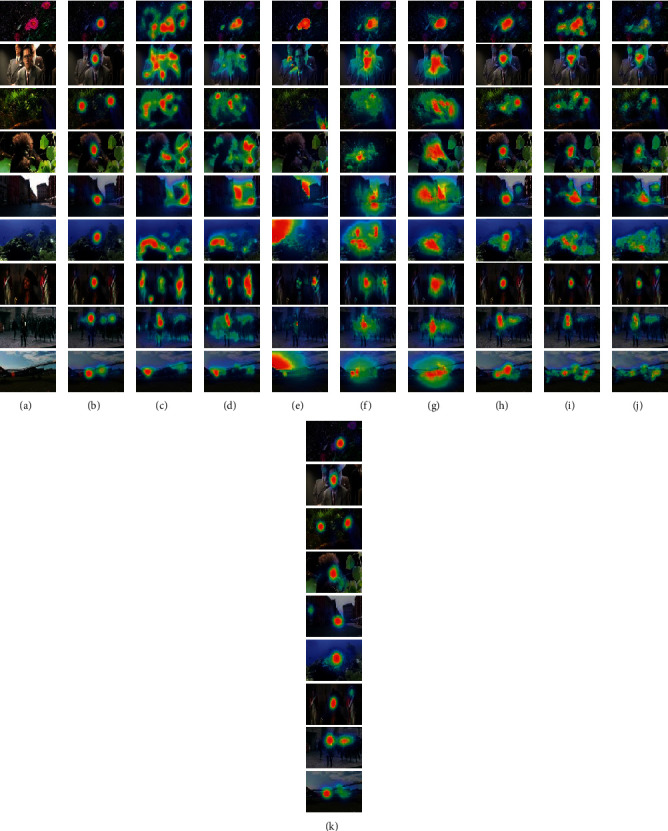
The results of various saliency models. (a) RGB. (b) GT. (c) Itti. (d) GBVS. (e) QFT. (f) Fang. (g) Qi. (h) DeepFix. (i) ML-net. (j) DVA. (k) Proposed.

For further illustration, Figure 2 shows some RGB-D saliency prediction examples for the models. The examples clearly show the computed performance of the proposed deep autoencoder in predicting the RGB-D saliency maps, which are more similar to their corresponding saliency density maps. In contrast, the saliency maps predicted using the other saliency models are significantly less consistent with their corresponding saliency density maps. In particular, the proposed deep autoencoder obtains high saliency values for people, objects, faces, and other predominant cues.

### 3.4. Model Ablation Study

We investigate various types of deep autoencoders from several aspects to shed more light on the proposed deep autoencoder, objectively evaluate the contribution of different networks in the proposed deep autoencoder against the two datasets, and evaluate the performance in terms of the CC, KLDiv, AUC, and NSS. To this end, we devised prediction performance comparison models, namely, *A*, *B*, and *C*. In model *A*, the deep autoencoder is without the disparity channel network. In model *B*, the deep autoencoder is without the prior learning subnetwork. In model *C*, the deep autoencoder is without the convolutional feature learning subnetwork.  Table 2 summarizes the prediction performances of models *A*, *B*, and *C*, including that of our model. The results demonstrate that the prediction performance of the saliency model improves when combining the color and disparity channel networks. Furthermore, it can be concluded that the prediction performance can be enhanced by optimally combining the prior learning and the convolutional feature learning subnetworks. In summary, the predictions obtained by comprehensively employing the different networks are found to be complementary, and the complete deep autoencoder can obtain more accurate saliency maps.

### 3.5. Analysis of Some Failure Cases

**Figure 3:**
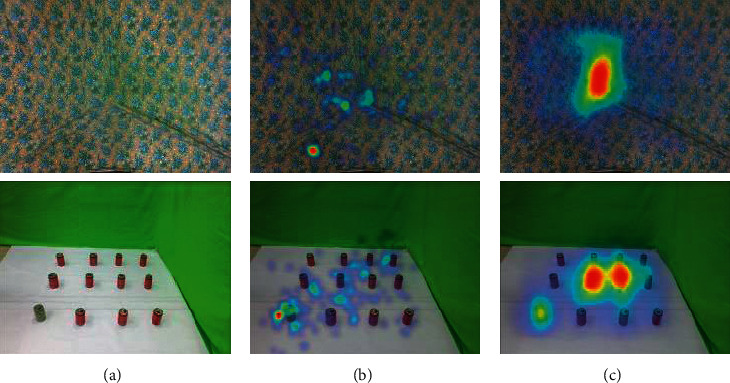
Some failure cases. (a) RGB. (b) Ground-truth. (c) Proposed.

**Figure 4:**
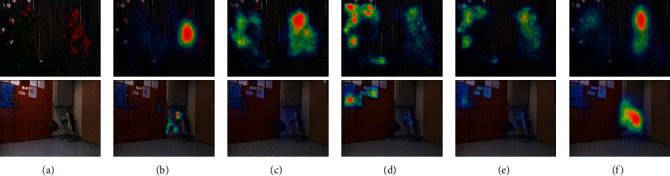
Some failure cases. (a) RGB. (b) GT. (c) DeepFix. (d) ML-net. (e) DVA. (f) Proposed.

Figures 3 and 4 show some typical failure cases. When there is no definite object in the RGB-D image attracting attention, human eye attention is inclined to be directed at the visual center. The proposed autoencoder fails to predict the same. In Figure 4 note that the prediction performances of the DeepFix, ML-net, and DVA, which are also based on CNNs, are not better than that of the proposed autoencoder when it comes to the RGB-D images.

## 4. Conclusion and Future Work

To reduce the semantic gap between model saliency prediction and human behavior, this work presents a first-of-its-kind deep multimodal fusion autoencoder for an accurate saliency prediction of RGB-D images. The main novelty of this study is the disparity channel network, which was specifically designed to boost the saliency prediction performance. Furthermore, the model optimally learns a combination of features extracted from a prior learning subnetwork and a convolutional feature learning subnetwork and applies it to predict the saliency maps. The effectiveness of each component was validated through extensive evaluations. The quantitative and qualitative comparisons with other models on two benchmark datasets indicate the efficiency and effectiveness of our deep autoencoder for the saliency prediction of RGB-D images.

In the future, we plan to design more effective saliency prediction models based on another deep multimodal fusion autoencoder and offer a deep investigation into the advantages of depth cues for RGB-D image saliency prediction.

## Figures and Tables

**Table 1:** The evaluation results of various saliency models.

Datasets	Criteria	Itti	GBVS	QFT	Fang	Qi	DeepFix	ML-net	DVA	Proposed
NUS	CC	0.341	0.396	0.163	0.333	0.371	0.4322	0.446	0.4549	0.5310
	KLDiv	1.457	1.374	1.795	1.560	1.505	1.8138	1.780	2.4349	1.2323
	AUC	0.788	0.824	0.682	0.795	0.806	0.7699	0.766	0.7236	0.8501
	NSS	1.236	1.441	0.568	1.209	1.357	1.6608	1.821	1.7962	2.1195

NCTU	CC	0.449	0.533	0.292	0.542	0.595	0.7974	0.696	0.6834	0.8034
	KLDiv	0.738	0.619	0.893	0.674	0.616	1.3083	0.900	1.1045	0.3593
	AUC	0.753	0.789	0.698	0.806	0.816	0.8650	0.835	0.8035	0.8671
	NSS	0.978	1.184	0.695	1.264	1.373	1.8575	1.588	1.5546	1.8405

**Table 2:** The prediction performances of models *A*, *B*, and *C*, as well as our proposed autoencoder.

Datasets	Criteria	Model *A*	Model *B*	Model *C*	Proposed
NUS	CC	0.5220	0.5227	0.5097	0.5310
	KLDiv	1.2538	1.3408	1.5606	1.2323
	AUC	0.8353	0.8351	0.7841	0.8501
	NSS	2.1198	2.1727	2.1301	2.1195

NCTU	CC	0.7607	0.8043	0.7967	0.8034
	KLDiv	0.3900	0.4152	0.3869	0.3593
	AUC	0.8552	0.8641	0.8618	0.8671
	NSS	1.7348	1.8914	1.8227	1.8405

## Data Availability

Two publicly available benchmark datasets were utilized: the NUS-3D Saliency dataset (denoted as NUS) and the NCTU-3D Fixation dataset (denoted as NCTU).
